# Impact of computerised physician order entry (CPOE) on the incidence of chemotherapy-related medication errors: a systematic review

**DOI:** 10.1007/s00228-021-03099-9

**Published:** 2021-02-23

**Authors:** Suresh Kumar Srinivasamurthy, Ramkumar Ashokkumar, Sunitha Kodidela, Scott C. Howard, Caroline Flora Samer, Uppugunduri Satyanarayana Chakradhara Rao

**Affiliations:** 1 Department of Pharmacology, Ras Al Khaimah College of Medical Sciences, Ras Al Khaimah Medical and Health Sciences University, Ras Al Khaimah, United Arab Emirates; 2 Cancer Services Business Informatics, Helen Diller Family Comprehensive Cancer, University of California, San Francisco (UCSF), San Francisco, CA USA; 3 The University of Tennessee Health Science Center, Memphis, TN USA; 4 Department of Acute and Critical Care, College of Nursing, University of Tennessee Health Science Center, Memphis, TN USA; 5 Division of Clinical Pharmacology and Toxicology, Faculty of Medicine, University of Geneva, Geneva, Switzerland; 6 Research Platform for Pediatric Onco-Hematology, Department of Paediatrics, Gynaecology and Obstetrics, University of Geneva, Geneva, Switzerland

**Keywords:** Medication errors, CPOE, Chemotherapy, Prescription, Patient safety

## Abstract

**Purpose:**

Computerised prescriber (or physician) order entry (CPOE) implementation is one of the strategies to reduce medication errors. The extent to which CPOE influences the incidence of chemotherapy-related medication errors (CMEs) was not previously collated and systematically reviewed. Hence, this study was designed to collect, collate, and systematically review studies to evaluate the effect of CPOE on the incidence of CMEs.

**Methods:**

A search was performed of four databases from 1 January 1995 until 1 August 2019. English-language studies evaluating the effect of CPOE on CMEs were selected as per inclusion and exclusion criteria. The total CMEs normalised to total prescriptions pre- and post-CPOE were extracted and collated to perform a meta-analysis using the ‘meta’ package in R. The systematic review was registered with PROSPERO CRD42018104220.

**Results:**

The database search identified 1621 studies. After screening, 19 studies were selected for full-text review, of which 11 studies fulfilled the selection criteria. The meta-analysis of eight studies with a random effects model showed a risk ratio of 0.19 (95% confidence interval: 0.08–0.44) favouring CPOE (I^2^ = 99%).

**Conclusion:**

The studies have shown consistent reduction in CMEs after CPOE implementation, except one study that showed an increase in CMEs. The random effects model in the meta-analysis of eight studies showed that CPOE implementation reduced CMEs by 81%.

**Supplementary Information:**

The online version contains supplementary material available at 10.1007/s00228-021-03099-9.

## Introduction

Medication error (ME) is defined as any preventable event that may cause or lead to inappropriate medication use or patient harm while the medication is in the control of the health care professional, patient, or consumer. Such events may be related to professional practice, health care products, procedures, and systems, including prescribing, order communication, product labelling, packaging, nomenclature, compounding, dispensing, distribution, administration, education, monitoring, and use [1]. ME is the ‘inappropriate use of a drug that may or may not result in harm’, and any harm occurring as a result of a ME is termed an adverse drug event (ADE) [2]. It is estimated that one-third of all hospital adverse events are attributed to ADEs; thus, drug safety significantly determines patient safety [3]. The World Health Organization (WHO) aptly launched in 2017 the third Global Patient Safety Challenge, ‘Medication Without Harm’ to reduce avoidable medication errors in all countries by over 50% by 2022 [4].

MEs in oncology have a greater impact on patient lives as cancer patients are vulnerable, and many chemotherapeutic agents exhibit a narrow therapeutic index [5]. The Institute of Safe Medication Practices has placed cancer chemotherapeutics at the top of the list of high-alert medications in acute, ambulatory, and long-term care settings [6]. MEs, such as dosing calculation errors, contribute significantly to the burden of ADEs, resulting in increased morbidity and mortality with additional economic impacts [7].

Chemotherapy-related medication errors (CMEs) affect 1–3% of oncology patients and occur in all phases of drug use, compromising safety [8, 9]. Previously, we reported rates of CMEs during the prescription, preparation, dispensing, and administration phases were 0.1–24.6%, 0.4–0.5%, 0–0.03%, and 0.02–0.1% of the total orders, respectively [10]. The prescription phase is highly vulnerable to the occurrence of errors that are largely preventable via strategies such as the implementation of computerised prescriber (or physician) order entry (CPOE). CPOE has shown promising results in preventing CMEs and improving safety in patients receiving complex chemotherapy regimens [11]. Although there are several reports on the utility of CPOE in improving patient safety in a chemotherapy setting, to our knowledge, no reports have collated and systematically reviewed the extent to which CPOE influences the occurrence of CMEs. Hence, this study was designed to collect, collate, and systematically review studies to evaluate the impact of CPOE implementation on the incidence of CMEs.

## Methods

### Search strategy

A search was performed using keywords such as ‘computerised physician order entry’, ‘computerised provider order entry’, ‘computerised prescriber order entry’, ‘CPOE and chemotherapy’, and ‘chemotherapy medication errors’. Using this search strategy (**Supplementary Material for Search Tree**), we explored the Medline, Web of Science, Agency for Healthcare Research and Quality, and Cumulative Index to Nursing and Allied Health Literature databases from 1 January 1995 to 1 August 2019. Three authors (SKS, RA, and SK) independently searched each database. The data collected from all databases were combined and screened for any duplication of records. Subsequently, the abstracts of all the relevant studies were reviewed for eligibility. During the full-text screening, the cited references were further scrutinised for relevance. This systematic review conformed to the Preferred Reporting Items for Systematic Reviews and Meta-Analyses (PRISMA) guidelines for reporting [12].

### Eligibility criteria

The inclusion criteria were as follows: (a) studies evaluating the effect of CPOE on CMEs, (b) studies providing numbers of CMEs with respect to the total number of prescriptions pre-and post-CPOE implementation, and (c) publications only in the English language. Studies evaluating multiple interventions and without non-intervention groups were excluded. Discrepancies regarding article inclusion and exclusion were resolved by discussion among all the screening and reviewing authors. Where necessary, the authors of the original reports were contacted for data on prescriptions for the analysis.

### Data extraction, quality assessment, and statistical analysis

Two reviewers (SKS and RA) extracted the data (Table 1). Using 13 different criteria adopted from previous reports, quality assessments were performed on all the studies included in the review [13, 14]. The criteria included reporting clearly described objectives, errors, error definitions, error categories, denominators, methodologies, settings, sample size calculations, reliabilities, validities, assumptions, limitations, and ethics committee approval. The quality scores were used to determine the overall applicability and impact of the studies, as well as to define the eligibility of studies for the meta-analysis. The extracted outcome data (i.e. risk ratios estimated using the Mantel–Haenszel method for the occurrence of CMEs pre- and post-CPOE) were collated to calculate the pooled estimates. A meta-analysis (random effects model) was performed using the ‘meta’ package in the R statistical software version 3.6.2 [15, 16]. Variance estimation (tau-squared) for the distribution of the true effect sizes was calculated using the DerSimonian–Laird estimator [17]. Publication bias was visualised using a funnel plot.

**Table 1:** Studies amalgamated to estimate the effect of CPOE on chemotherapy-related medication errors

Study	Kim et al. 2006	Voeffray et al. 2006	Markert et al. 2009	Collins et al. 2011	Cheng et al. 2012*	Elsaid et al. 2013	Meisenberg et al. 2014	Sanchez Cuervo et al. 2015	Aziz et al. 2015	Wang et al. 2017*	Chung et al. 2018*
Hospital setting (type)	Academic medical centre	University hospital	Clinical service centre based in University	Tertiary care hospital paediatric division	University-affiliated medical centre	Urban multidisciplinary hospital	Acute care hospital with cancer institute	University hospital	Tertiary hospital	University hospital	Community owned safety net health system Three sites: LBJ, BT, SC
Country	USA	Switzerland	Germany	USA	Taiwan	USA	USA	Spain	Pakistan	China	USA
Patient setting	NA	Inpatient and outpatient	Inpatient and outpatient	Inpatient and outpatient	Inpatient and outpatient	Inpatient and outpatient	Inpatient and outpatient	Inpatient and outpatient	NSP	NSP	Inpatient and outpatient
Population	Paediatric	Adult	Adult	Paediatric and adult	Paediatric and adult	Paediatric and adult	NSP	NSP	NSP	NSP	NSP
Pre-CPOE prescription mode	Paper based	Pre-formatted hand written	Manual	Paper order form	Hand written	Hand written	Hand written ^**#**^	Hand written	Paper based order	Hand written	Preprinted order
Data collection	Retrospective	Retrospective and Prospective**	Prospective	Retrospective	Retrospective	Retrospective	Retrospective	Prospective	Prospective	Prospective**	Retrospective and Prospective**
Study time period—pre-CPOE (days)	241	450	365	720	306	900	365	121	180	NA	NSP
Study time period—post-CPOE (days)	296	630	730	180	368	840	NAC	90^##^	180	NA	180
Number of prescriptions analysed Pre-CPOE	1255	9400	10,885	412	8417	28,560	2216	721	5514	21,589	60
CMEs (event rate%; 95% CI) Pre- CPOE	157 (12.5; 10.7–14.4)	141 (15.0; 12.8–17.4)	930 (8.5; 8.0–9.1)	39 (9.5; 6.8–12.7)	281 (3.3; 3.0–3.7)	507 (1.8; 1.6–1.9)	772 (34.8; 32.9–36.9)	270 (37.4; 33.9–41.1)	134 (2.4; 2.0–2.9)	562 (2.6; 2.4–2.8)	60 (100; 94–100)
Number of prescriptions analysed Post-CPOE	1116	978	22,005	126	10,273	43,206	5142	748	3765	5950	40
CMEs (event rate%; 95% CI) Post-CPOE	163 (14.6; 12.6–16.8)	6 (0.6; 0.2–1.3)	1636 (7.4; 7.1–7.8)	4 (3.2; 0.9–7.9)	41 (0.4; 0.3–0.5)	340 (0.8; 0.7–0.9)	118 (2.3; 1.9–2.7)	9 (1.2; 0.6–2.3)	10 (0.3; 0.1–0.5)	34 (0.6; 0.4–0.8)	10 (25; 12.7–41.2)
Relative risk reduction or increase (%) Post-CPOE	17^###^	96	13	66	88	56	93	97	89	77	75

*Studies were not included in meta-analysis. **Data collection not clearly explained

^#^Hand written data was used as pre-CPOE: the study had a hand-written phase (12 months), preprinted forms (12 months), run in time 2 months CPOE phase (NAC). ^##^Post-CPOE data collected after 5 years of implementation. ^###^Increase in CMEs

*BT*, Ben Taub General Hospital affiliated with Baylor College of Medicine; *CI*, confidence interval; *CPOE*, computerised prescription order entry; *LBJ*, Lyndon B. Johnson General Hospital affiliated with the University of Texas Medical School; *NAC*, not able to calculate precise intervals; *NA*, not available; *NSP*, non-specified; *SC*, Smith Clinic; *USA*, United States of America

## Results

### Study selection

**Fig. 1:**
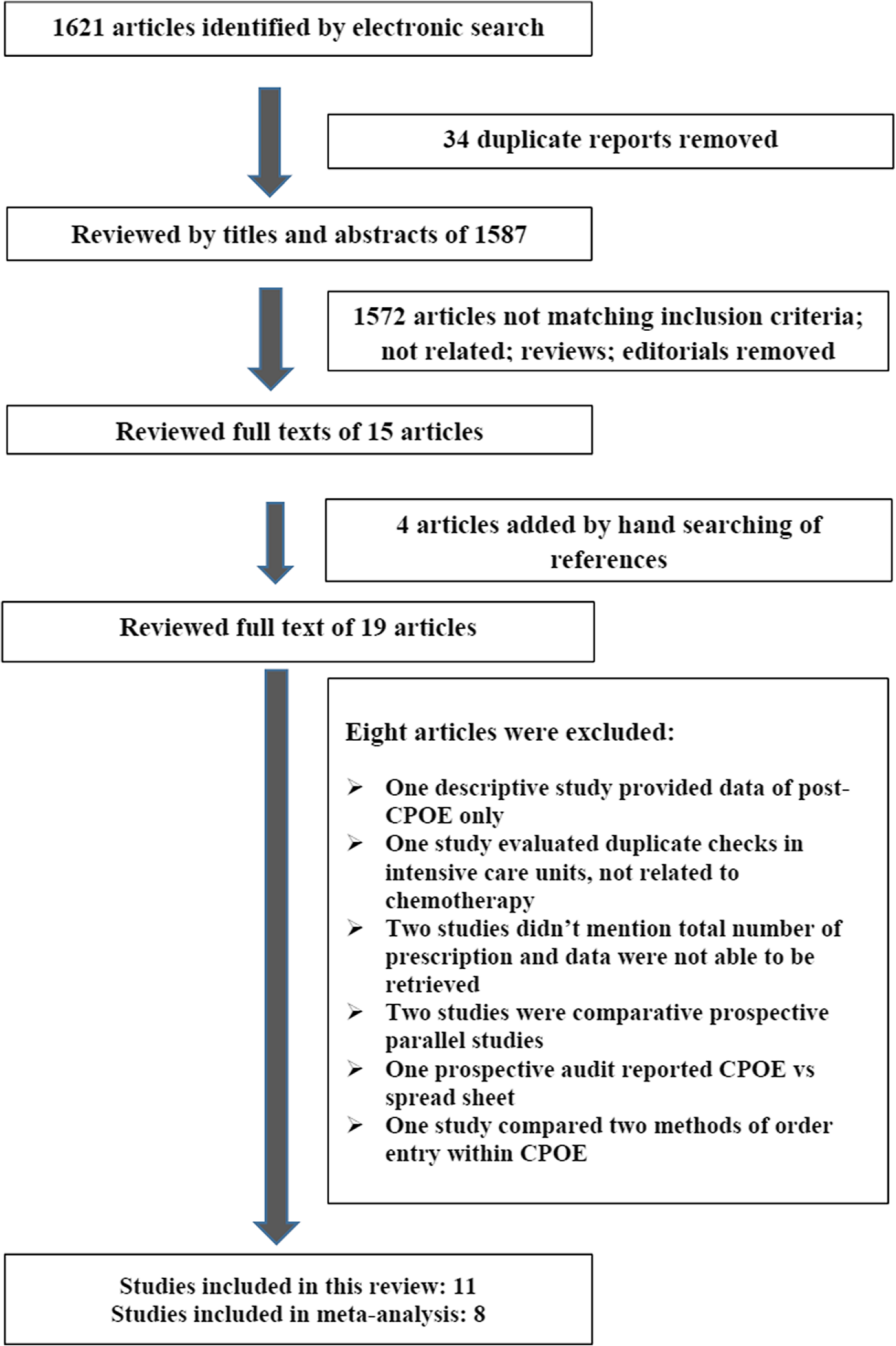
PRISMA diagram of the literature search conducted

Of the 1621 studies identified by the search, 34 duplicate and 1572 unrelated reports were removed. The remaining 15 full-text articles plus four additional articles cited in these papers were reviewed. Among the 19 studies, eight did not meet the eligibility criteria [18–25]. Thus, 11 studies were eligible and received a consensus as shown in Fig. 1. Among the excluded studies that did not meet inclusion criteria, one descriptive study provided data of post-CPOE alone [18]. Another study evaluated duplicate checks in a non-chemotherapy setting [19]. Two studies did not mention total number of prescriptions, and we were unable to retrieve the data by correspondence with the authors [20, 21]. Two studies were comparative prospective parallel studies [22, 23]. One prospective audit reported CPOE vs. spread sheet [24]. One study compared two methods of order entry within CPOE [25].

### Study characteristics

All 11 studies were published between 2006 and 2018 and had a single-arm design in which the CPOE pre-and post-implementation phases were compared [26–36]. Among these, five studies were from the USA, three from Europe, and three from Asian countries. The extracted data on CME events and the study parameters are shown in Tables 1 and 2. The average CME event rate (number of events/total number of prescriptions, in percentage) pre-CPOE implementation was 20.7% (range: 1.8–100%) and was reduced to 5.12% (range: 0.26–25.0%) post-CPOE. Nine studies have shown statistically significant reductions in CMEs post-CPOE compared to pre-CPOE (Table 2). Significant reductions in CMEs related to medical devices, adjuvants, infusion rates, and routes of administration have been documented [26, 27]. However, in one of the included studies, there was a statistically significant increase in a specific type of CME—i.e. a mismatch between orders and treatment plans post-CPOE [RR 5.4 (CI 3.1–9.5)]—that affected the total CME events [26].

**Table 2:** Characteristics of CPOE used in studies with their impact on chemotherapy-related medication errors and clinical implications

Study	Kim et al. 2006	Voeffray et al. 2006	Markert et al. 2009	Collins et al. 2011	Cheng et al. 2012*	Elsaid et al. 2013	Meisenberg et al. 2014	Sanchez Cuervo et al. 2015	Aziz et al. 2015	Wang et al. 2017*	Chung et al. 2018*
CPOE system	RxTFC Pharmacy Information System; GE Medical Systems with FMEA	Inbuilt system (File Maker Pro)	Electronic chemotherapy ordering and prescription (eCOP) system	Siemens Invision system with HFMEA	Inbuilt system with HFMEA	Siemens Medical Solutions’ + (CDSSs) + (EDDSs), + bar-code point-of-care medication administration system	Beacon system	ONCOWIN version 8.0	Inbuilt system with CDSS	complete prescription audit system (CPAS) with HFMEA	Beacon EPIC systems
Phase of treatment investigated	P,D,A,T	P	P,A	P,A	P,D,A,T	P	P	P,A	P,D,A,Pr	P,D,A,T	P
Chemotherapy medications evaluated/reported^a^	NA	NA	PAC, 5FU, VIO, CYT, CET, ETO, CIS, GEM, OX	MTX, MP, HU	NA	BEV, CAR, CET, CIS, CYC, CYT, DOC, DOX, ETO, MTX, PAC, RTX, TRA, VIN, 5FU, OX	CYC, DOX, VIN, P, CIS	CYC, RTX, MTX	DOX, CYC 5FU, VIB, BLE, DA OX, RTX	NSP	NSP
Inferential statistical analysis performed	Yes	Yes	No	Yes	Yes	Yes	Yes	Yes	No	Yes	Yes
User satisfaction reported	No	No	No	No	No	No	No	No	Yes	Yes	Yes
Clinical implications of errors reported	NSP	Major errors recorded^b^ Pre-CPOE 19% of CMEs Post-CPOE 0% of CMEs	SAE incidence/order/year reported^c^ Pre-CPOE 0.8% of CMEs Post-CPOE: 0.76% of CMEs	Harmful CMEs^d^ Pre-CPOE 33% of CMEs Post-CPOE: 0% of CMEs	Quantitative data not reported	Life threatening CMEs requiring medical interventions Pre-CPOE 15% of CMEs Post-CPOE 0% of CMEs	Harmful errors^e^ Pre-CPOE 4.2% of total prescriptions Post-CPOE 0.1% of total prescriptions	NSP^f^	Fatal and serious CMEs of pharmacy interventions Pre-CPOE 36.5% of CMEs Post-CPOE 20% of CMEs	Adverse reactions (errors)^g^ Pre-CPOE 2.6% of total prescriptions Post-CPOE 0.6% of total prescriptions	NSP

*Studies not included in the meta-analysis

^a^Primary drug evaluated or reported errors as specific examples

^b^Major errors had a potential effect on patients, such as involving the type of medical device used (e.g. infusion bag or syringe) or route of administration, whereas minor errors indicated the volume or type of infusion solutions

^c^Serious adverse events (SAEs) included unexpected deaths, chemotherapy-induced extravasations, unexpected referrals to the intensive care unit, unscheduled operations, and any serious, undesirable events

^d^Drug dose and/or schedule errors such as temozolomide orders that deviated from the standard regimen

^e^Harmful errors were of potential to cause harm

^f^The study described dosing errors, which could have clinical impact, accounting for 37% of all errors and was eliminated post-CPOE

^g^The clear distinction between ME and adverse events was not explained

*A*, administration; *CI*, confidence interval; *CPOE*, computerised prescription order entry; *CPAS*, complete prescription audit system; *eCOP*, electronic chemotherapy ordering and prescription; *CDSS*, clinical decision support system; *D*, dispensing; *FMEA*, failure mode and effect analysis; *EDDSs*, electronic drug dispensing systems; *HFMEA*, healthcare failure mode and effects analysis; *NAC*, not able to calculate precise intervals; *NA*, not available; *NS*, not significant; *NSP*, non-specified; *P*, prescription or order; *Pr*, preparation; *SAE*, serious adverse events; *T*, transcription; *USA*, United States of America

Drugs: *BEV*, bevacizumab; *CAR*, carboplatin; *CET*, cetuximab; *CIS*, cisplatin; *CYC*, cyclophosphamide; *CYT*, cytarabine; *DOC*, docetaxel; *DOX*, doxorubicin; *ETO*, etoposide; *GEM*, gemcitabine; *MTX*, methotrexate; *PAC*, paclitaxel; *RTX*, rituximab; *TRA*, trastuzumab; *VIN*, vincristine; *VIO*, vinorelbine; *5FU*, 5 flurouracil; *OX*, oxaliplatin; *P*, prednisolone; *VIB*, vinblastine; *BLE*, bleomycin; *DA*, dacarbazine; *MP*, mercaptopurine; *HU*, hydroxy urea; *PC*, carboplatin + paclitaxel; *AC*, Adriamycin + cyclophosphamide

The different types of CPOE used in the studies are listed in Table 2. One of the studies reported complete elimination of all CMEs with adequate training and acclimatisation of personnel over a period of 5 years [33]. Two studies did not provide inferential statistics; however, the number of events pre- and post-CPOE differed significantly (Table 2, *P*<0.05) [28, 34]. The average quality score of the included studies was 7.2 with 95% CI (6.08–8.12) (**Supplementary Table** **1**).

Healthcare Failure Mode and Effects Analysis (HFMEA) or Failure Mode and Effects Analysis (FMEA) showed reductions in CMEs post-CPOE in four of the included studies [26, 29, 30, 35]. HFMEA strategies have been adopted in conjunction with CPOE with additional alerting modules for pathological conditions such as renal and liver dysfunction to execute dose modifications accordingly [30]. CPOE was also shown to reduce CMEs when complemented with supporting systems such as complete prescription audit systems (CPASs) [35] and clinical decision support systems (CDSSs) [34].

The maximum numbers of prescriptions were evaluated by two studies having a quality score of 8 [28, 31]. Markert et al. demonstrated a reduction in CMEs (including patient data errors) during the prescription phase from 8.5% to 7.4% following CPOE [28]. In the same report, CMEs in outpatients decreased from 4% to 2.8% following CPOE, whereas for inpatients, it remained unchanged (4.4% vs. 4.7%) [28]. The study showed that the presence of a multidisciplinary clinical service centre (CSC) prevented 99.92% of all CMEs reaching the patients. The patients’ risk of experiencing a CME was estimated to be 0.13% of the total treated patients. However, the incidence of serious adverse events (SAEs) per patient per year was reported at 7.5% and 7.4%, respectively, pre- and post-CPOE (Table 2) [28]. Elsaid et al. have also reported error rates during three phases: pre-implementation (30 months), implementation (32 months), and post-implementation (28 months) [31]. The prescribing errors that were prevented per 1000 doses during pre-implementation, implementation, and post-implementation were 17.8, 9.1, and 7.9, respectively. The study showed that CPOE reduces CMEs with the highest effect being on dosing calculation errors, which were reduced by 94% [31].

Meisenberg et al. reported CMEs from three sequential patterns of prescription orders: handwritten (30.6%), preprinted (12.6%), and CPOE (2.2%) [32]. The harmful CMEs among the reported CMEs also showed a statistically significant decrease from handwritten (4.2%) to preprinted (1.5%) and CPOE (0.1%). This retrospective study involved analysing every 10th order of the handwritten and preprinted orders and every fifth order during the CPOE phase [32]. Collins et al. reported a 69% reduction in prescription errors for oral chemotherapy medication within 6 months post-CPOE [29]. In addition to reporting CMEs, one study reported improvement in dispensing and administration time with the use of CPOE. The mean administration time was reduced from 132 min (pre-CPOE) to 112 min (post-CPOE) [34].

User satisfaction was surveyed in three studies [34–36]. Aziz et al*.* showed that residents, consultants, and pharmacists perceived CPOE to be user friendly, while nurses perceived it as not user-friendly [34]. High satisfaction scores were reported for all end users by Wang et al. [35]. Chung et al., however, reported higher user satisfaction in one centre with the moderate dissatisfaction in two centres attributed to the problems in acclimatisation with the new system [36]. However, overall, no difference in satisfaction scores before and after CPOE was reported [36].

Three studies were prospectively designed to collect data from the prescription validation process while implementing CPOE [28, 33, 34], while in five studies, the data were collected as part of the routine quality assurance process and analysed retrospectively [26, 29–32]. The data collection methods were not clearly explained in three studies [27, 35, 36].

Seven studies reported clinical implications of CPOE on the occurrence of serious or fatal events among CMEs [27–29, 31, 32, 34, 35]. The major, fatal, or serious adverse events (SAEs) that ranged from 0.8 to 36.5% of CMEs pre-CPOE were reduced from 0% to 20% post-CPOE (Table 2). Adverse events with clinical implications were completely eliminated in three studies [27, 29, 31]. In one study, the SAE numbers remained unchanged pre- and post-CPOE; however, the SAE incidence per order per year decreased marginally from 0.8% to 0.76% [28]. Serious and fatal events reduced post-CPOE in another study from 36.5% to 20% [34].

### Meta-analysis

**Fig. 2:**
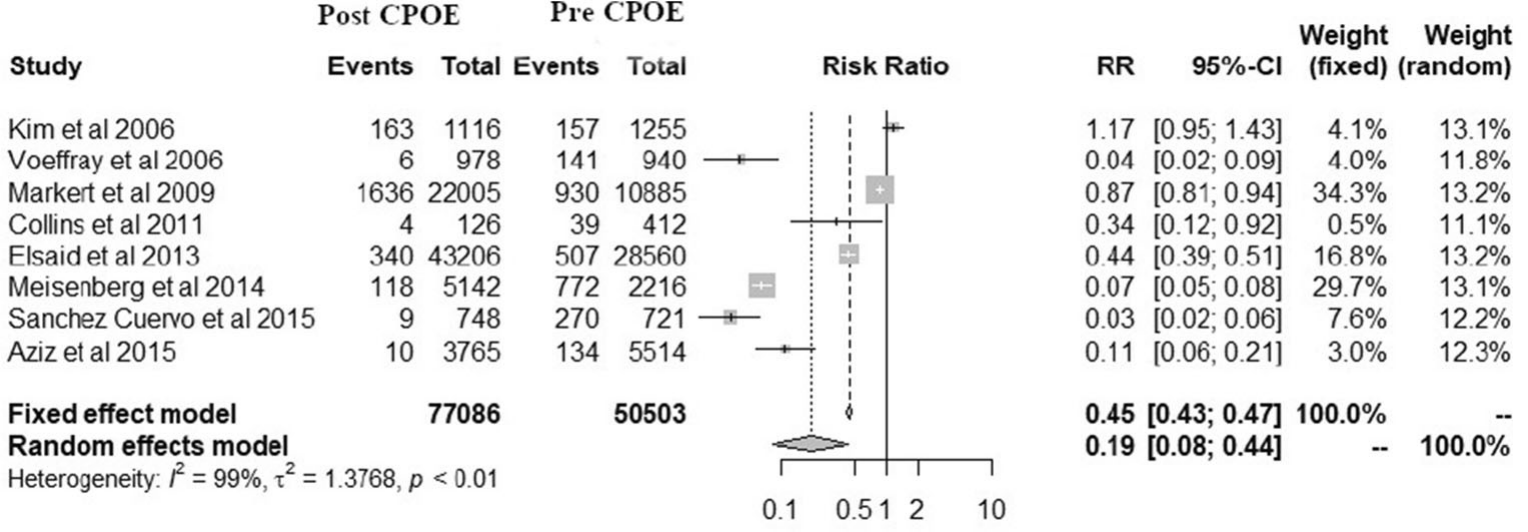
Forest plot of the studies reporting chemotherapy related medication errors pre- and post-CPOE implementation. The vertical line represents ‘line of no effect’. The post-CPOE data is shown on the left side of the vertical line, whereas the right side represents pre-CPOE data. *X*-axis represents relative risks

The total number of CMEs reported with respect to the total number of prescriptions pre- and post-CPOE implementation was collated in the meta-analysis (Fig. 2). Studies with quality scores below the lower limit of 95% CI (≤ 6) were excluded from the meta-analysis [30, 35, 36]. Among these, one case study reported data from ‘selected’ prescriptions only that might have incorporated bias [36]. The favourable pooled effect with CPOE implementation resulted in an 81% reduction in CMEs. A pooled risk ratio (RR) of 0.19 (95% CI: 0.08–0.44) was observed favouring CPOE (random effects model, I^2^ = 99%) implementation (Fig. 2). The funnel plot was asymmetrical, indicating the presence of bias due to heterogeneity among the included studies (Supplementary Figure 1). Further investigations on asymmetry were not conducted as the number of studies included in the meta-analysis was below 10.

## Discussion

To our knowledge, this systematic review represents the first effort to amalgamate available data on CME occurrence pre-and post-CPOE implementation (Table 1). Our analysis showed that CPOE implementation resulted in a significant reduction in CMEs (81%), indicating that it is a valuable strategy that can be used to reduce CMEs (Table 1; Fig. 2). The beneficial effects of CPOE have been previously reported in a descriptive study [37]. Similarly, CPOE implementation was shown to reduce 76% of MEs in all hospitalised patients [38] and by 85% in the intensive care unit patients [39].

Although CPOE implementation is valuable, it requires constant monitoring and training, especially during the initial implementation [18, 26]. The study by Kim et al. highlighted the need to link computerised treatment protocols with drug protocols to prevent CMEs [26]. The statistically significant increase in the non-matching of orders to treatment plans post-CPOE emphasised the need for proper preparation and constant surveillance, even post-CPOE [26]. Meisenberg et al. described CPOE (Beacon system)–related errors, such as unintended re-escalation of doses if the wrong cycle was copied while creating additional cycles; confusion while adding or deleting drug in a regimen without changing the title; retention of supportive medications even after the deletion of chemotherapy drugs; chances of overdosing when treatment days were reduced as the data was automatically updated for future cycles; and inappropriate omission of drugs if the prescriber forgot to sign the order [32].

Nevertheless, CPOE systems have evolved to offer solutions for such problems, e.g. adoption of HFMEA strategies which included additional alerting modules for pathological conditions such as liver dysfunction, enabling the execution of dose modifications, accordingly [30]. The US Joint Commission on Accreditation of Healthcare Organizations also advocates the use of HFMEA to improve patient safety [40]. CPOE also functioned well with complementary supporting systems such as CPASs [35] and CDSSs [36]. CPOE combined with an integrated CDSS, especially with artificial intelligence, could be an effective approach to medication safety [41, 42]. Importantly, CDSS modules customised to chemotherapy settings should be compliant with chemotherapy protocols, dose calculations, and dose adjustments. Furthermore, they must have provisions in place for alerts at the crucial juncture of prescribing and mandatory items to ensure completeness of the prescription process [43].

The data on the impact of CPOE on clinical outcomes were included in seven studies (Table 2) [27–29, 31, 32, 34, 35]. The magnitude of the reduction in the CMEs was translated to a reduction in adverse events, as shown by three studies where major, serious, and fatal adverse events were completely eliminated post-CPOE [27, 29, 31]. This is in agreement with a meta-analysis of reports from hospital-based settings which observed a nearly 50% reduction in preventable ADRs and medication errors (RR = 0.46; 95% CI: 0.35–0.60) upon CPOE implementation [44]. Aziz et al. showed a decrease in fatal and serious events post-CPOE [34]. However, Markert et al. reported an unchanged total number of SAEs pre- and post-CPOE, and a marginal decrease in SAE incidence per order per year [28]. The harmful errors were normalised to the total orders in the report by Meisenberg et al. [32]. However, there was no proper distinction between errors and adverse reactions in the findings of Wang et al. [35]. Thus, future studies designed to report CMEs should also include data on SAEs, which have important clinical implications.

Furthermore, CPOE improved the completeness of documentation and user satisfaction in outpatient oncology settings [45]. Thus, with respect to medication safety, CPOE is a structural asset in sensitive healthcare settings, including oncology and onco-haematology units. CPOE implementation should involve a multidisciplinary team of physicians, nurses, pharmacists, clinical pharmacologists, and information technology (IT) professionals. CPOE systems can eliminate MEs completely and can yield good results in an optimum time of 5 years after complete adaptation [33].

Our review should be viewed in light of the following strengths and limitations. The included studies were from different parts of the globe; hence, the results have generalisability and applicability. Most of the studies included were single-centre studies except for one study [36], which compromised the external validity (Table 1). Although single-arm designs have several limitations, they provide preliminary evidence of the effect in most scenarios. The data reported from the included studies of chemotherapy settings also included MEs from supportive care therapy, which are used along with chemotherapy. Nevertheless, all studies used different CPOE systems and in different settings; thus, the external validity of studies should be gauged. Our meta-analysis showed high heterogeneity (Fig. 2) due to the differences in hospital settings, reporting time periods, patient populations, sample sizes, data collection methods, and CPOE types (Table 1) and the definitions used to demarcate CMEs among the limited number of studies included. The high heterogeneity observed among studies limited their comparability that is commonly seen in studies reporting MEs as reported earlier [10].

The average quality scores of the studies included in this review (*n*=11) and meta-analysis (*n*=8) were 7.2 and 8.0, respectively, out of 13. Although most of the studies were poor in reporting reliability and validity measures, those excluded from meta-analysis scored poorly on study objectives, error definitions, error categories, and data collection methodologies. We also propose the implementation of and adherence to comprehensive checklist/uniform standards while reporting ME so that studies would be comparable.

In conclusion, a systematic review of 11 studies showed consistent reduction of CMEs after the implementation of CPOE. However, one study showed an increase in CMEs, which was attributed to improper preparation and acclimatisation. CPOE implementation reduced CMEs by 81% in a meta-analysis of eight studies. Thus, CPOE could be an effective strategy for limiting CMEs, provided that multidisciplinary approach to training and acclimatisation is provided.

## Supplementary information

ESM 1 (DOCX 163 kb)

## Data Availability

The study protocol was registered with PROSPERO CRD42018104220. All other relevant data mentioned in the article are provided in the supplementary material.
