# Supplementary material for: Impact of computerised physician order entry (CPOE) on the incidence of chemotherapy-related medication errors: a systematic review
Source: Eur J Clin Pharmacol. 2021 Feb 23;77(8):1123–31. doi: 10.1007/s00228-021-03099-9 (PMC8275496; doi:10.1007/s00228-021-03099-9)
Supplement: Supplementary file 1 — (DOCX 163 kb) [file 228_2021_3099_MOESM1_ESM.docx]

Impact of Computerised Physician Order Entry (CPOE) on the Incidence of Chemotherapy-related Medication Errors: A Systematic Review

Suresh Kumar S^1^; Ramkumar A^2^; Sunitha K^3^, Scott C Howard^4^, Samer CF^5^, Uppugunduri CRS^6^.

^1^Department of Pharmacology, RAK College of Medical Sciences, RAK Medical & Health Sciences University, Al Qusaidat, Ras Al Khaimah, United Arab Emirates

^2^Program Manager for Cancer Services Business Informatics, Helen Diller Family Comprehensive Cancer, University of California, San Francisco (UCSF), San Francisco, California, United States of America

^3^The University of Tennessee Health Science Center, Memphis, TN, United States of America (USA).

^4^Department of Acute and Critical Care, College of Nursing, University of Tennessee Health Science Center, Memphis, Tennessee, United States of America

^5^Division of Clinical Pharmacology and Toxicology, Faculty of Medicine, University of Geneva, Geneva, Switzerland.

^6^Research Platform for Pediatric Onco-Hematology; Department of Paediatrics, Gynaecology and Obstetrics; University of Geneva; Geneva, Switzerland

**Correspondence:**

Uppugunduri S Chakradhara Rao

Research Platform for Paediatric Onco-Hematology; Department of Paediatrics, Gynaecology and Obstetrics; University of Geneva; Geneva, Switzerland. Email: [Chakradhara.Uppugunduri@unige.ch](mailto:Chakradhara.Uppugunduri@unige.ch)

**Search tree: Present draft of search strategy to be used for at least one electronic database, including planned limits, such that it could be repeated**

((Computerized physician order entry) AND oncology)OR((Computerized physician order entry) AND cancer)OR

((Computerized physician order entry) AND chemotherapy)OR((Computerized physician order entry) AND (chemotherapy medication errors))OR((Computerized physician order entry) AND (chemotherapy medication error)) OR((Computerized Provider order entry) AND oncology)OR((Computerized Provider order entry) AND cancer)OR((Computerized Provider order entry) AND chemotherapy)OR((Computerized Provider order entry) AND (chemotherapy medication errors))OR((Computerized Provider order entry) AND (chemotherapy medication error)) OR((Computerized Prescriber order entry) AND oncology)OR((Computerized Prescriber order entry) AND cancer)OR((Computerized Prescriber order entry) AND chemotherapy)OR((Computerized Prescriber order entry) AND (chemotherapy medication errors))OR((Computerized Prescriber order entry) AND (chemotherapy medication error))OR((CPOE) AND oncology)OR((CPOE) AND cancer)OR((CPOE) AND chemotherapy)OR((CPOE) AND (chemotherapy medication errors))OR((CPOE) AND (chemotherapy medication error))

**Supplementary Table 1:** Quality assessment of all the studies reviewed. The score of one indicates mentioned and zero indicates not mentioned in the studies. The quality assessment criteria were as adopted from Ghaleb *et al.* 2006 and Alsulami *et al.* 2013 [13, 14]

| **Criteria used for quality scoring** | Kim *et al.* 2006 | Voeffray *et al.* 2006 | Markert *et al.* 2009 | Collins *et al.* 2011 | Cheng *et al.* 2012 | Elsaid *et al.* 2013 | Meisenberg *et al.* 2014 | Aziz *et al.* 2015 | Sanchez Cuervo *et al.* 2015 | Wang *et al.* 2017 | Chung *et al.* 2018 |
| --- | --- | --- | --- | --- | --- | --- | --- | --- | --- | --- | --- |
| 1. Aim/objective of the study is mentioned | 1 | 1 | 1 | 1 | 0 | 1 | 1 | 1 | 1 | 1 | 1 |
| 2. Definition of what constitutes medication error | 1 | 1 | 0 | 1 | 1 | 1 | 1 | 1 | 1 | 0 | 0 |
| 3. Error is mentioned | 1 | 1 | 1 | 1 | 1 | 1 | 1 | 1 | 1 | 1 | 1 |
| 4. Error category is defined | 1 | 1 | 1 | 1 | 1 | 1 | 1 | 0 | 1 | 0 | 0 |
| 5. Clearly defined Denominator is mentioned | 1 | 1 | 1 | 1 | 1 | 1 | 1 | 1 | 1 | 1 | 1 |
| 6. Data collection method | 1 | 0 | 1 | 1 | 1 | 1 | 1 | 1 | 1 | 0 | 0 |
| 7. Setting is mentioned | 1 | 1 | 1 | 1 | 1 | 1 | 1 | 1 | 1 | 1 | 1 |
| 8. Sampling and calculation of sample size described | 0 | 0 | 0 | 0 | 0 | 0 | 0 | 0 | 1 | 0 | 0 |
| 9. Reliability measures | 0 | 0 | 1 | 0 | 0 | 1 | 0 | 0 | 0 | 0 | 0 |
| 10. Measures in place to ensure that results are valid | 0 | 0 | 0 | 0 | 0 | 0 | 0 | 0 | 0 | 0 | 0 |
| 11. Limitations of study listed | 1 | 1 | 0 | 1 | 0 | 0 | 1 | 1 | 1 | 0 | 1 |
| 12. Mention of any assumptions made | 0 | 0 | 0 | 0 | 0 | 0 | 1 | 0 | 0 | 0 | 0 |
| 13. Ethics approval | 0 | 0 | 1 | 0 | 0 | 0 | 0 | 0 | 0 | 0 | 0 |
| **Total Score** | **8** | **7** | **8** | **8** | **6** | **8** | **9** | **7** | **9** | **4** | **5** |

**
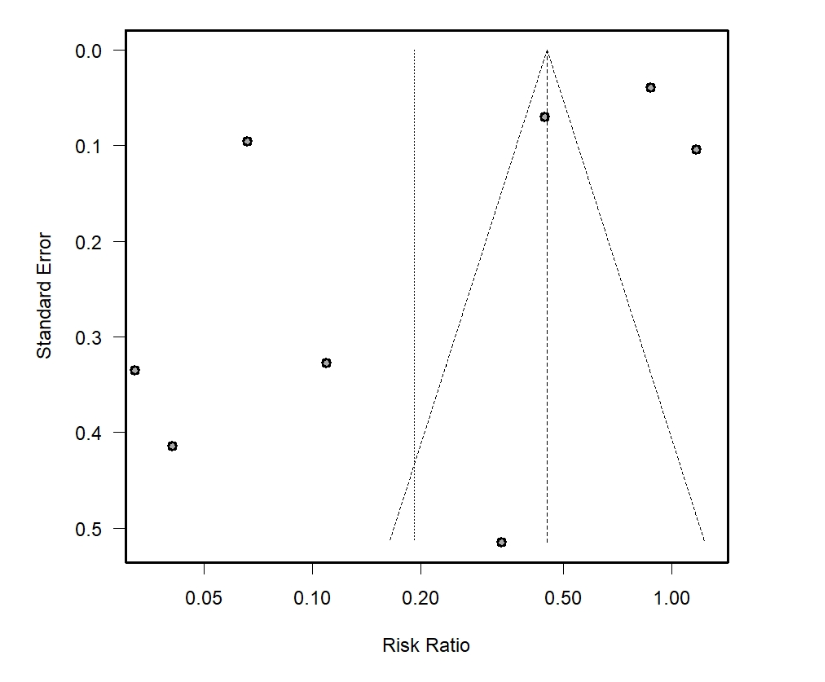

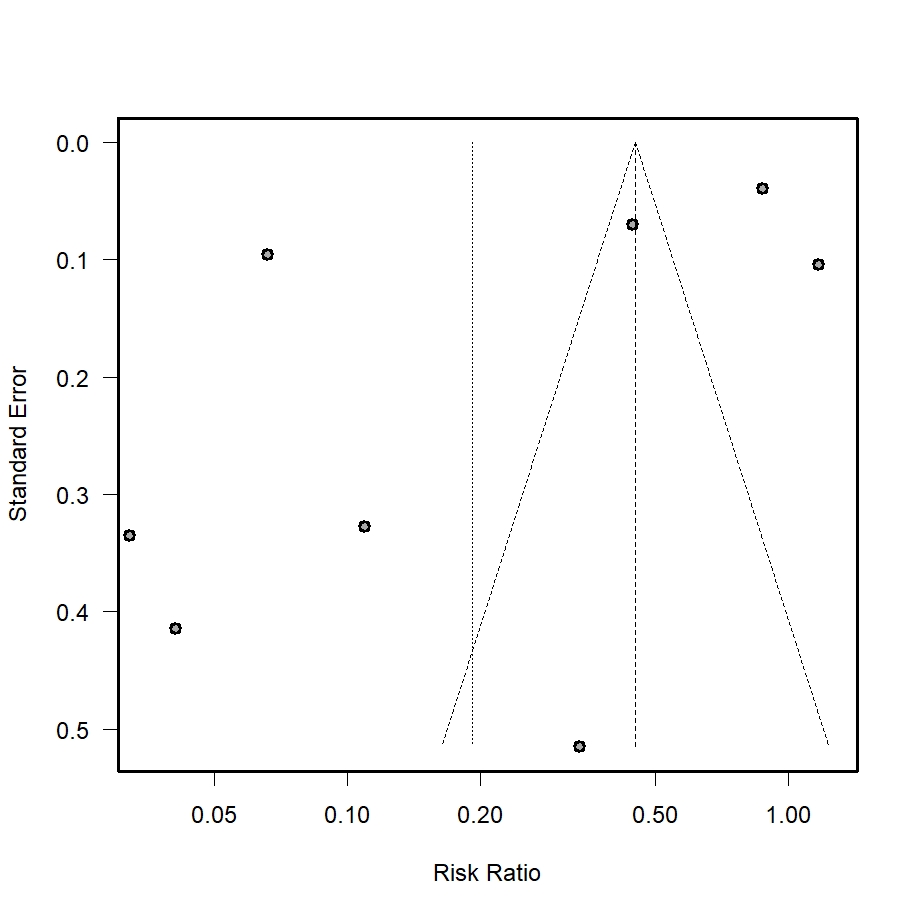
**

**
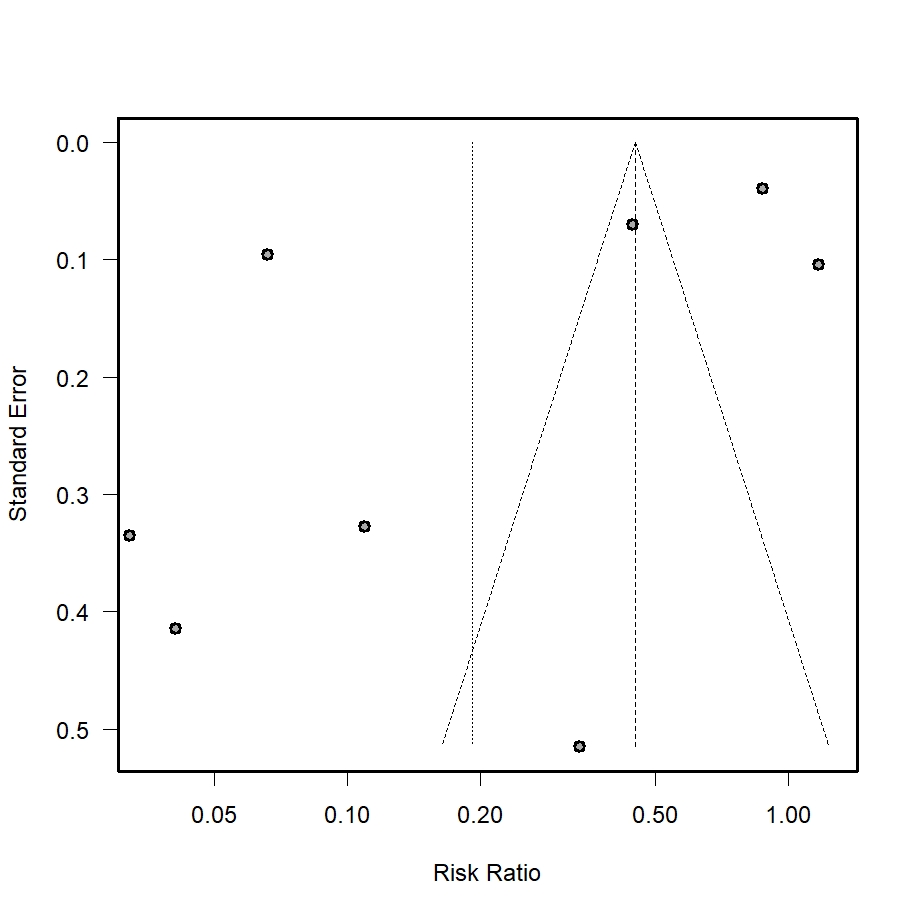
**

**Supplementary Figure 1**: Funnel plot of data from meta-analysis of eight studies
